# Pneumococcal conjugate vaccination at birth in a high-risk setting: No evidence for neonatal T-cell tolerance

**DOI:** 10.1016/j.vaccine.2011.05.065

**Published:** 2011-07-26

**Authors:** Anita H.J. van den Biggelaar, William Pomat, Anthony Bosco, Suparat Phuanukoonnon, Catherine J. Devitt, Marie A. Nadal-Sims, Peter M. Siba, Peter C. Richmond, Deborah Lehmann, Patrick G. Holt

**Affiliations:** a Telethon Institute for Child Health Research, Centre for Child Health Research, University of Western Australia, PO Box 855, West Perth WA 6872, Australia; b Papua New Guinea Institute of Medical Research, PO Box 60, Goroka, EHP 441, Papua New Guinea; c School of Paediatrics and Child Health, University of Western Australia, Princess Margaret Hospital for Children, GPO Box D184, Perth WA 6840, Australia

**Keywords:** Immunisation, Newborn, Pneumococcal conjugate vaccine, Safety, Immunogenicity

## Abstract

Concerns about the risk of inducing immune deviation-associated “neonatal tolerance” as described in mice have restricted the widespread adoption of neonatal vaccination. The aim of this study was to demonstrate the immunological feasibility of neonatal pneumococcal conjugate vaccination (PCV) which could potentially protect high-risk infants in resource poor countries against severe pneumococcal disease and mortality in the early critical period of life. Papua New Guinean infants were randomized to be vaccinated with the 7-valent PCV (7vPCV) at birth, 1 and 2 months (neonatal group, *n* = 104) or at 1, 2 and 3 months of age (infant group, *n* = 105), or to not receive 7vPCV at all (control group, *n* = 109). Analysis of vaccine responses at 3 and 9 months of age demonstrated persistently higher type-1 (IFN-γ) and type-2 (IL-5 and IL-13) T-cell responses to the protein carrier CRM_197_ and IgG antibody titres to 7vPCV serotypes in children vaccinated with 7vPCV according to either schedule as compared to unvaccinated children. In a comprehensive immuno-phenotypic analysis at 9 months of age, no differences in the quantity or quality of vaccine-specific T cell memory responses were found between neonatal vaccinations versus children given their first PCV dose at one month. Hospitalization rates in the first month of life did not differ between children vaccinated with PCV at birth or not. These findings demonstrate that neonatal 7vPCV vaccination is safe and not associated with immunological tolerance. Neonatal immunisation schedules should therefore be considered in high-risk areas where this may result in improved vaccine coverage and the earliest possible protection against pneumococcal disease and death.

## 1 Introduction

Findings by Medawar and colleagues [1] in the 1950s that infant mammals fail to reject allografts expressing antigens they have been exposed to in foetal and neonatal life gave rise to the concept of neonatal tolerance. A series of landmark studies in 1996 [2–4] collectively demonstrated that rather than deletional tolerance, this phenomenon represented ‘immune deviation’ involving selective activation of T helper 2 (Th2) immunity by functionally immature neonatal antigen presenting cells (APC), resulting in attenuation of the class of immunity (Th1) that is central to graft rejection. The process may represent the transient carry-over of a functional phenotype which dominates the innate immune system during foetal development, the purpose of which is to attenuate the production at the foetal-maternal interface of Th1 cytokines which are highly toxic towards the placenta [5,6].

These findings have raised legitimate concerns regarding the potential risks of neonatal vaccination against pathogens, namely that vaccination during neonatal life when antigen presenting cells retain their foetal-like Th2-selectivity, may inadvertently compromise the capacity to develop effective and persistent Th1-polarised immunity. This concern has been supported by data from neonatal vaccination studies in mice [7,8] and studies in humans demonstrating a general type-2 polarisation of T-cell memory to certain vaccines other than Bacillus Calmette-Guérin (BCG) following infant priming [9–14]. As a result this issue continues to cast a shadow of doubt over the possibility of immunizing neonates. This is of a particular concern in neonates in resource poor countries as they especially are subjected to a high mortality rate from vaccine preventable diseases. Moreover, neonatal immunisation is likely to improve overall vaccine coverage as mothers are more likely to come into contact with health services around the time of delivery [15,16]. Hence, neonatal immunisation might be more favourable than infant immunisation if proven to be safe and equally immunogenic.

In 2008 alone, an estimated 0.9 million newborns died of sepsis or pneumonia [17]: a number that could be reduced by neonatal vaccination strategies. To study the immunological feasibility of pneumococcal vaccination in human newborns, we directly compared immune responses to PCV in newborns and older infants in Papua New Guinea (PNG): continuing our previous published work on early vaccine responses at 3 months of age [18], memory T-cell responses to the vaccine protein carrier CRM_197_ were immuno-phenotyped and compared between the three groups at 9 months of age by means of *in vitro* cytokine response assays for all study participants, complemented with microarray studies comparing genome-wide T-cell related gene expression in a randomly chosen subgroup of children in the neonatal compared to the infant group (*n* = 25 per group). In addition, aiming to address the functionality of the memory T-cell responses, PCV-serotype specific IgG antibody titres were determined and studied in relation to *in vitro* vaccine protein carrier specific cytokine responses.

## 2 Materials and methods

### 2.1 Study design and participants

The study area and population recruitment in PNG have been described elsewhere [18]. Briefly, pregnant women were recruited at the antenatal clinic of Goroka Hospital and in villages located within an hour's drive of Goroka town. Inclusion criteria were the intention to remain in the study area for at least 2 years, a birth weight of at least 2000 g, no acute neonatal infection and no severe congenital abnormality.

Newborns (*n* = 313) were randomised to receive 7vPCV (Prevnar^®^, Wyeth) within 3 days of birth, 1 and 2 months (neonatal group, *n* = 104), at 1, 2 and 3 months (infant group and corresponding with the PNG national expanded program on immunisation age, *n* = 105), or to receive no 7vPCV (control group, *n* = 109). In accordance with the PNG national expanded program on immunisation, all study children received BCG (birth); oral polio vaccine (neonatal, 1, 2 and 3 months), Hepatitis B (neonatal, 1 and 3 months), a combined *Haemophilus influenzae* type b, diphtheria, tetanus, whole cell pertussis vaccine (TETRActHib) (1, 2 and 3 months), and measles vaccine (6 and 9 months).

A data safety monitoring board (DSMB) was established and was immediately advised of any serious adverse events and of all adverse events 3-monthly.

This trial is registered at ClinicalTrials.gov under registration number NCT00219401 (http://clinicaltrials.gov/ct2/show/NCT00219401).

### 2.2 Ethical considerations

Assent was sought from women and their partners at the time of recruitment. Written informed consent was obtained after delivery and before enrolment of the newborn child. Ethical approval was obtained from the PNG Medical Research Advisory Committee and the Princess Margaret Hospital Ethics Committee in Perth, Australia.

### 2.3 Serum collection and isolation of peripheral mononuclear cells (PBMC)

At 3 and 9 months of age, venous blood samples (1–2.5 ml) were collected into empty 2-ml tubes (serum) and 10-ml sterile tubes containing 100 IU preservative-free heparin (PBMC). Samples were centrifuged within 2 h to separate serum/plasma and aliquots were stored at −20 °C. PBMC were isolated from the remaining heparin tube cell pellet by centrifugation over a Ficoll-Hypaque gradient (Lymphoprep, Alexis-Shield, Oslo, Norway) and cryo-preserved in 50% heat-inactivated (HI) foetal calf serum (FCS) and 7.5% DMSO. Cells were kept under liquid nitrogen vapour phase conditions during storage at IMR, transport to and storage at the Telethon Institute of Child Health Research (ICHR).

### 2.4 *In vitro* PBMC cultures

PBMC were cultured in duplicate in 96-wells plates (1 × 10^6^ cells/ml) in medium (RPMI/5% HI-inactivated human AB serum) (Pharmacia Australia Pty. Ltd., Sydney, Australia) or stimulated with CRM_197_ (kindly provided by former Wyeth Pharmaceuticals, USA) (2.5 μg/ml), Tetanus Toxoid (TT; CSL, Victoria, Australia) (0.5 lf/ml), measles lysate (kindly provided by Steven Wesselingh and Diane Webster, Macfarlane Burnet Institute for Medical Research, Melbourne, Australia) (4 × 10^5^ particles/ml) and phytohemagglutinin (PHA; Remel Europe Ltd., Kent, UK) (positive control, 1 μg/ml). Supernatants were collected after 96 h (48 h for PHA). Due to low blood volumes, sufficient PBMC for *in vitro* CRM_197_ experiments (including negative and positive controls) were available for 198 children at 3 months (neonatal 68; infant 68; control 62) [18] and 222 children at 9 months (neonatal 74; infant 76; control 72); 132 children (neonatal 48; infant 46; control 38) had *in vitro* CRM_197_ data available for both time points. For 9 months samples, *in vitro* stimulations with TT and measles lysate could be performed for 99 (neonatal, *n* = 40; infant, *n* = 28; control, *n* = 31) and 113 children (neonatal, *n* = 43; infant, *n* = 33; control, *n* = 37), respectively.

### 2.5 Cytokine protein detection

Cytokine levels were measured using an in-house multiplex assay. Briefly, microspheres (MagPlex, Luminex^®^, USA) coupled to azide-free primary antibodies against IL-5, IL-6, IL-9, IL-10, IL-12, IL-13 and TNFα (Becton Dickinson, USA) and IFN-γ and IL-1β (eBioscience, USA) in PBS-BN (PBS + 1% BSA + 0.05% Sodium Azide, pH 7.4) (1 × 10^6^ beads/ml) were plated onto 96-well plates (Costar^®^, USA) (50 μl/well), followed by the addition of cytokine standards, quality controls, or samples. Standards were diluted in culture media and assay buffer (PBS + 1%BSA), and quality controls and samples in assay buffer. A magnetic bead separator was used to wash the plates. After addition and incubation with biotinylated-secondary antibodies, plates were incubated with streptavidin-PE (Becton Dickinson, USA) (1:1000 in assay buffer), washed and assay buffer was added before reading on a Bio-Plex Suspension Array System (BIO-RAD, USA). Samples with concentrations below the detection limit were given the value corresponding to half the lowest concentration that could be detected in this set of samples.

### 2.6 Microarray studies

In a time course experiment a 72 h *in vitro* culture period was found to best capture the expression of both early and late CRM_197_-induced memory T-cell genes (target genes: IL-2, IL-4, IL-5, IL-9, IL-13, IL-17, IFNγ, CXCL10, GZMB, LIF and Foxp3; data not shown). Total RNA was extracted from non-stimulated and CRM_197_-stimulated PBMC (25 neonatal; 25 infant) using TRIzol (Invitrogen) followed by RNeasy (Qiagen). For each microarray experiment, 150 ng of pooled RNA of 5 subjects belonging to the same study arm was labelled and hybridized to Human Gene 1.0 ST microarrays (Affymetrix), employing standardized protocols and reagents from Affymetrix (total of 20 microarrays). Microarray data were pre-processed in Expression Console software (Affymetrix) using the probe logarithmic intensity error algorithm, then imported into the R environment (version 2.9.1; www.r-project.org) for further analysis [19]. Significance analysis of microarrays (SAM) [20] was employed to identify genes that were significantly modulated in response to CRM_197_ stimulation and compare CRM_197_-specific gene expression profiles between the two groups: to account for multiple testing, SAM uses an internal procedure to estimate the false discovery rate (FDR) [21]. DAVID Bioinformatics Resources 6.7 was used to identify functional clusters amongst induced genes [22]. The microarray data are available in the Gene Expression Omnibus repository (www.ncbi.nlm.nih.gov/projects/geo/) under the accession number GSE25263.

### 2.7 Quantitative real time PCR

Reverse transcription was performed using the Qantitect kit (Qiagen, USA) according to the manufacturer's protocol with oligo-dT (Promega, USA) and Superasin (GeneWorks, Australia). Intron-spanning primers for IL-2, IL-2Ra, IL-4, IL-5, IL-9, IL-13, IL-17F, IL-17RB, IFNγ, CXCL10, GZMB, LIF and Foxp3 were obtained from http://pga.mgh.harvard.edu/primerbank and designed in-house using Primer Express Software (Applied Biosystems, USA). Reverse-transcribed RNA samples were diluted 1/5 and quantitated by real-time PCR using QuantiTect SYBR Green Master Mix (Qiagen) on the ABI PRISM 7900HT (Applied Biosystems). Copy numbers were determined by 10-fold serial dilutions of plasmid standards and normalized to the reference gene eukaryotic translation elongation factor 1 alpha 1 (EEF1A1).

### 2.8 Pneumococcal capsular polysaccharide ELISA

Serum IgG antibodies to PCV serotypes (4, 6B, 9V, 14, 18C, 19F and 23F) were measured using a WHO standardised ELISA [23]. Briefly, microtitre plates (Greiner, Germany) were coated with capsular polysaccharide antigens for 5 h at 37 °C. Serum samples were added after overnight absorption with 10 μg/ml cell wall polysaccharide and 5 μg/ml serotype 22F. The WHO reference serum 89SF (FDA, Bethesda, MD) was pre-absorbed with 10 μg/ml cell wall polysaccharide. Goat anti-human IgG conjugate (Biosource, CA, USA) and pnPP substrate (Sigma, USA) were used for detection. Each plate contained a high and low in-house quality control serum to assess intra- and inter-assay variations.

### 2.9 Statistical analysis

Statistical analysis of data other than the microarray studies were performed using SPSS 15.0. To compare categorical variables, the Pearson chi-square and Cramer's V were calculated for 2 × 2 and 2 × 3 tables respectively. Mann–Whitney tests or Kruskal–Wallis tests were used to compare continuous data in two or three groups, respectively. Cytokine responses were log_10_-transformed and data presented as geometric means (GM) ± the standard error of the geometric means (SEGM). Spearman rank correlation analysis was performed to study correlations between 7vPCV serotype-specific IgG antibody titres and CRM_197_-specific cytokine responses. For all analysis, test outcomes were considered to be significant if the *p*-value was smaller or equal to 0.05.

## 3 Results

### 3.1 Study numbers

Population characteristics for the children at the time of enrolment have been described elsewhere [18]. Of the 313 children enrolled at birth, 255 were eligible for follow-up and data analysis at 9 months of age (neonatal 81; infant 91; control 83): of the 58 children lost to the study at 9 months, parental consent was withdrawn for 32 children (neonatal, *n* = 14; infant, *n* = 7; control, *n* = 11); 10 children were lost to follow-up due to migration out of the study area (neonatal, *n* = 3; infant, *n* = 1; control, *n* = 6); 15 children were excluded from analysis due to protocol violations (neonatal, *n* = 4; infant, *n* = 3; control, *n* = 8); and one child died (infant group). Sufficient PBMC for *in vitro* CRM_197_ stimulations were available for 222 children (neonatal 74; infant 76; control 72) at 9 months of age; for 132 children cell culture data at both 3 and 9 months of age were available (neonatal 48; infant 46; control 38).

### 3.2 Neonatal and infant PCV vaccination induces persistent memory responses

The capacity of either vaccination schedule to induce persistent immune memory was assessed by determining vaccine responses at 3 months of age when children in the infant group were still to receive a 3rd dose of PCV, and at 9 months of age, long after completion of the different immunisation schedules.

**Fig. 1:**
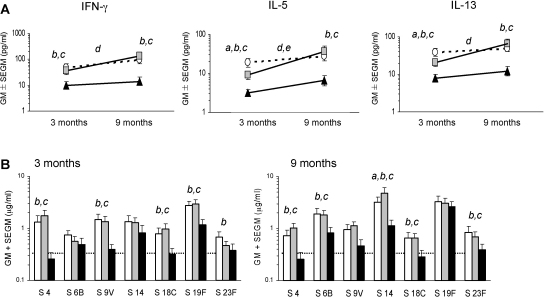
CRM_197_-specific type-1 and type-2 recall responses at 3 and 9 months of age. (A) Geometric means and standard errors of geometric means of CRM_197_-induced IFN-γ (T helper 1), IL-5 and IL-13 (T helper 2) protein responses at 3 and 9 months (○, neonatal *n* = 48; ■, infant, *n* = 46; ▴, control, *n* = 38). The letters indicate significant differences (*p* < 0.05) for each time point between (*a*) the neonatal vs. infant group, (*b*) the neonatal vs. control group and (*c*) the infant vs. control group, and within groups for 3 compared to 9-month responses for the (*d*) infant and (*e*) control group (no statistical differences were found within the neonatal group). (B) Pneumococcal vaccine serotype-specific IgG antibody responses in the neonatal (white bar, *n* = 44), infant (grey bar, *n* = 40) and control group (black bar, *n* = 32). A level of 0.35 μg/ml or more is considered a clinical correlate of protection against invasive pneumococcal disease [35].

In Fig. 1A, *in vitro* type-1 (IFN-γ) and type-2 (IL-5 and IL-13) T-cell memory cytokine responses to the vaccine carrier protein CRM_197_ are shown for 132 study children with data available at both 3 and 9 months of age: type-1 and type-2 CRM_197_ responses were found to be higher in PCV-vaccinated children compared to unvaccinated children at both 3 and 9 months of age, which confirms that both 7vPCV immunisation schedules induced persistent T-cell memory responses. The higher Th2-recall response at 3 months of age in the neonatal compared to the infant group (IL-5, *p* = 0.044; IL-13, *p* = 0.051) [18], was no longer apparent at 9 months of age. There was no evidence that co-immunisation with BCG at birth influenced CRM_197_-induced T-cell responses at 3 months [18] or 9 months of age (data not presented).

Accordingly, at both 3 and 9 months of age serum pneumococcal serotype-specific IgG antibody titres were found to be higher and exceed the assumed clinically protective level of 0.35 μg/ml for most PCV serotypes in children who had received 7vPCV compared to those who had not, confirming the induction of protective immune responses in both immunisation schedules (Fig. 1B).

### 3.3 Neonatal and infant vaccination induce identical recall responses

We next performed a comprehensive immuno-phenotypic analysis of the CRM_197_-specific T-cell memory response at 9 months to confirm that recall responses were similar under neonatal and infant immunisation schedules.

**Fig. 2:**
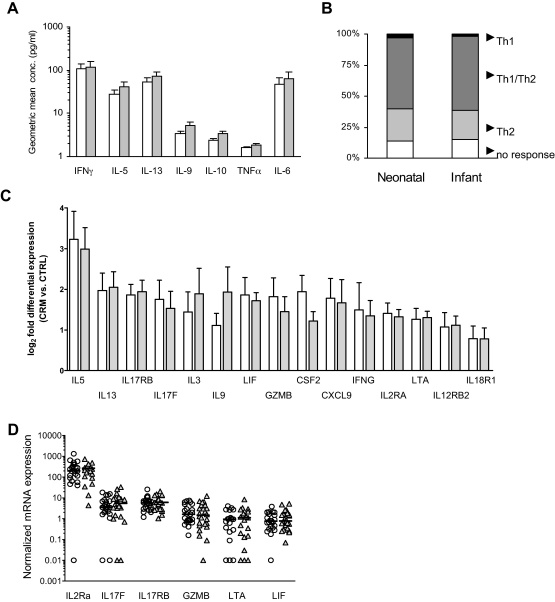
Immunophenotypic analysis of recall T cell responses at 9 months following neonatal or infant immunisation. (A) Background-adjusted protein cytokine responses to CRM_197_ in the neonatal (white bar; *n* = 65) and infant groups (grey bar; *n* = 62) at 9 months. (B) Proportion of children responding with an exclusive Th1 (IFN-γ) (black), mixed Th1/Th2 (IFN-γ, and IL-5 and/or IL-13) (dark grey), exclusive Th2 (IL-5 and/or IL-13) (light grey), or no protein cytokine response to CRM_197_ (positive response ≥ 4 times the background) (white). (C) Microarray-assessed log_2_-fold differential gene expression (mean and standard error of the mean) in CRM_197_-stimulated cells compared to that in un-stimulated cells in the neonatal (white bar, *n* = 5 × 5 pooled PBMC) and infant groups (grey bar, *n* = 5 × 5 pooled PBMC). (D) Quantitative RT-PCR based relative mRNA expression in CRM_197_-stimulated compared to that in un-stimulated PBMC in the neonatal (○, *n* = 20) and infant group (, *n* = 20), normalized against the constantly expressed gene eukaryotic translation elongation factor 1 a 1 (EEF1A1). CSF2, colony stimulating factor 2; CXCL9, Chemokine (C-X-C motif) ligand 9; GZMB, Granzyme B; IFNG, interferon-γ; Interleukin, IL; LIF, Leukemia inhibitory factor; LTA, lymphotoxin A.

Comparison of *in vitro* T-cell memory cytokine responses to the vaccine carrier protein CRM_197_ demonstrated that recall responses were characterized by a mixed pattern of type-1/type-2 responses and were comparable in the neonatal and infant groups (Fig. 2A), with only a minority of children displaying exclusively Th2 responses (Fig. 2B).

We next progressed to genome-wide expression profiling of T-cell memory responses in a subpopulation of randomly selected children (*n* = 25 per group). In the neonatal group *in vitro* CRM_197_ stimulation was found to result in a significant differential expression of 105 genes and in the infant group of 140 genes (78 mutual) (Supplementary Fig. 1A and Table 1). The expression levels of these CRM_197_ response genes did not differ between the two vaccination groups (Supplementary Fig. 1B) and this is illustrated in Fig. 2C for genes that are involved in T-helper cell differentiation or responsiveness. Since microarray data were based on pooled RNA samples and could be influenced by individual outliers, we performed quantitative RT-PCR analysis for a defined set of memory T-cell related genes in individual samples. In line with the microarray data, mRNA expression levels were similar in the two vaccination groups (Fig. 2D).

### 3.4 Neonatal immunisation induces functional type-1 and permissive type-2 responses

To address the functionality of the vaccine-induced type-1 response and the potential negative effect of type-2 responses to neonatal vaccination, we studied in the neonatal group correlations between CRM_197_-specific IFN-γ, IL-5 and IL-13 responses at age 3 months (1 month after the 3rd dose of PCV) and PCV serotype-specific IgG antibody titres at age 3 and 9 months. As demonstrated in Table 1, CRM_197_-IFN-γ responses at age 3 months correlated significantly with antibody titres at 9 months; this confirms the ability of neonatal immunisation to induce functional type-1 immunity. Furthermore, the positive associations between the Th2 response and circulating antibody titres at age 3 months suggest that Th2 responses do not negatively interfere with the induction of immunity, but rather facilitate responses, possibly by driving initial B-cell switching and proliferation.

### 3.5 Immunological safety of neonatal vaccination

**Fig. 3:**
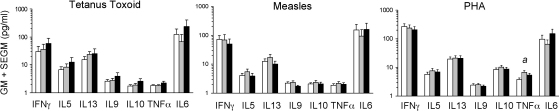
Bystander T cell cytokine responses. Background adjusted *in vitro* cytokine responses to tetanus toxoid (neonatal, *n* = 40; infant, *n* = 28; control, *n* = 31), measles lysate (neonatal, *n* = 43; infant, *n* = 33; control, *n* = 37) or phytohemagglutinin (PHA) (neonatal, *n* = 74; infant, *n* = 74; control, *n* = 72) at 9 months of age. (*a*) indicates significant difference in the neonatal compared to infant group.

One measure of demonstrating the safety of neonatal vaccination is excluding the possibility of any interference with cellular immune responses to expanded program of immunisation (EPI) vaccines or with normal maturation of the immune system. We have previously demonstrated that at 3 months of age type-1 and 2 cytokine responses to the concomitant vaccine antigens PPD (BCG), HbsAg (HepB) and TT (DTwP/Hib), and polyclonal T cell responses to PHA were similar in the 3 study groups [18]. Repeating this measure at 9 months of age for responses to TT and PHA as well as the later administered measles vaccine (1st dose at 6 months of age), cellular immune responses were again found to be similar in the three groups (except for higher PHA-TNFα responses in the infant than in the neonatal group, *p* = 0.004) (Fig. 3).

Hospitalization in the first month of life children did not differ between children in the neonatal vaccination group (1.3/1000 person days) compared to those who had not received a neonatal dose (3.0/1000 person days) (*p* = 0.18), indicating that neonatal vaccination did not impose an early health risk.

## 4 Discussion

In this study we have shown in human newborns at high risk of pneumococcal disease and death that both neonatal and infant PCV immunisation schedules successfully prime and induce persisting protective immune responses in these high-risk infants; that neonatal immunisation with PCV induces a similar type-1/type-2 memory response as vaccination starting at the current PNG EPI age of 1 month (which is a bit earlier than most schedules starting at 6 weeks of age in developing countries); and that vaccine-induced Th2 responses do not negatively interfere with the induction of immunity.

Our results are in disagreement with mouse studies showing that vaccination in early life induces skewed Th2 responses, with little development of sterilizing Th1 immunity. Although the primary response in neonatal mice appears to compromise both Th1 and Th2 cells [24], Th1 cells appear to undergo apoptosis in response to a secondary challenge while Th2 cells remain responsive [25,26]. To date, only a few human studies have reported on the effect of neonatal vaccination on T-cell development. Whereas Bacillus Calmette-Guérin induces adult-like Th1 responses when administered at birth [27–29], the capacity of other vaccines to induce type-1 responses is lower in neonates than in adults unless responses are enhanced by co-administered BCG [9,12–14]; however, the clear skewing towards Th2-type responses and elimination of Th1 responses that is observed in mice [30] is not readily apparent in human newborns. By comparing recall responses in infants that completed a 3-dose immunisation schedule starting either shortly after birth or after the neonatal period at the age of 1 month, we have been able to demonstrate that, in line with findings for BCG, neonatal immunisation with other vaccines such as this pneumococcal conjugate vaccine is safe and not associated with immune deviation.

Alongside the induction of competent Th1 responses, neonatal and infant PCV vaccination elicited comparable Th2 responses that, as illustrated by initial positive associations with vaccine antibody titres, were facilitating and not attenuating protective vaccine serotype-specific responses. Although DT- and CRM_197_-containing conjugate vaccines such as the PCV used in this study have been associated with vaccine interference [31], no evidence for this was found in our study. We therefore believe that the neonatal Th2 milieu does not pose more risks than vaccination schedules starting later in infancy and that the induction of Th2 responses is not an impediment to neonatal vaccination.

We found that serum IgG antibody titres varied according to pneumococcal serotype; this is a well-recognized phenomenon to both unconjugated and conjugated pneumococcal vaccines. Antibody titres might also be affected by carriage of pneumococcal serotypes commonly circulating in the community such as serotype 19F for which non-vaccinated children also showed high antibody titres. Moreover, 19F has been reported to be the least efficacious component of PCV [32], which may explain that in contrast to our findings for the other six PCV serotypes CRM_197_-IFN-γ responses at age 3 months did not correlate significantly with IgG antibody responses to 19F at 9 months.

A limitation of our neonatal vaccination trial was the small blood volume that could be obtained from young infants; this restricted the breadth and depth of immunological experiments that could be performed. Nevertheless, we have been able to perform and present a comprehensive immuno-phenotypic analysis of vaccine responses within the first nine months of infancy, including genome-wide microarray and RT-PCR experiments in addition to *in vitro* cell cultures and serum antibody responses measured at different time points. Since the aim of this trial was to demonstrate the safety and immunogenicity of neonatal PCV vaccination, the study was not powered to demonstrate any clinical benefit of neonatal PCV vaccination. However, our data strongly support larger randomized controlled trials to assess efficacy.

In conclusion, in humans neonatal vaccination with PCV induces similar T helper memory development as vaccination later in infancy: this finding challenges the paradigm described in mice that neonatal immunisation induces immune deviation-associated “neonatal tolerance”. Furthermore, our study highlights the importance of understanding the role of T helper cells in vaccine responses in paediatric populations, all the more so considering the expanding use of polysaccharide conjugate vaccines [33] and increasing interest in using vaccine adjuvants to enhance cellular immune responsiveness [34].

## Figures and Tables

**Table 1:** Spearman correlations for 3-month CRM_197_ responses and 3 and 9 month 7vPCV serotype-specific IgG antibody titres in the neonatal group.

	3-Month IgG						9-Month IgG					
	CRM_197_ response at 3 months						CRM_197_ response at 3 months					
	IFN-γ		IL-5		IL-13		IFN-γ		IL-5		IL-13	
	rho	*p*-Value	rho	*p*-Value	rho	*p*-Value	rho	*p*-Value	rho	*p*-Value	rho	*p*-Value
Serotype 4	0.375	0.004	0.459	<0.001	0.379	0.004	0.524	<0.001	0.183	0.181	0.175	0.202
6B	0.157	0.227	0.367	0.004	0.231	0.073	0.474	<0.001	0.072	0.614	0.161	0.259
9V	0.272	0.032	0.206	0.108	0.147	0.253	0.307	0.024	0.233	0.091	0.136	0.326
14	0.107	0.413	0.310	0.015	0.202	0.119	0.259	0.064	0.002	0.988	0.019	0.891
18C	0.352	0.005	0.245	0.057	0.189	0.144	0.294	0.032	0.160	0.253	0.081	0.565
19F	0.118	0.362	0.300	0.018	0.162	0.208	−0.008	0.956	0.012	0.934	−0.042	0.765
23F	0.420	0.001	0.491	<0.001	0.502	<0.001	0.271	0.050	0.154	0.272	0.138	0.326
