# Supplementary material for: Pneumococcal conjugate vaccination at birth in a high-risk setting: No evidence for neonatal T-cell tolerance
Source: Vaccine. 2011 Jul 26;29(33-19):5414–20. doi: 10.1016/j.vaccine.2011.05.065 (PMC3146700; doi:10.1016/j.vaccine.2011.05.065)
Supplement: Supplementary file 1 [file mmc1.doc]

**Supplementary Table 1. Functional clusters of CRM_197_**-induced genes

|  | **CRM_197_ vs. CTRL**  **log_2_-fold change** | |  |
| --- | --- | --- | --- |
|  | **Neonatal** | **Infant** |  |
| **Immune/inflammatory response** | |  |  |
| AICDA | 1.16 | 1.08 | b |
| C1S | 0.73 | 0.48 | b |
| CD14 | -1.41 | -1.24 | c |
| CD163 | -1.13 | -1.33 | c |
| CD274 | 0.78 | 0.83 | c |
| CISH | 0.98 | 0.98 | a |
| CSF2 | 1.71 | 1.13 | a |
| CTSW | 0.93 | 0.87 | a |
| CXCL9 | 1.45 | 1.41 | a |
| EXO1 | 1.01 | 0.95 | c |
| FABP4 | -0.99 | -1.20 | c |
| FGL2 | 0.65 | 0.90 | c |
| GBP4 | 0.78 | 0.79 | c |
| GBP5 | 0.92 | 0.87 | c |
| GBP6 | 1.22 | 1.61 | c |
| GNLY | 0.84 | 0.66 | b |
| GZMA | 1.03 | 0.99 | a |
| GZMB | 1.80 | 1.44 | a |
| HTR1A | 0.22 | 0.54 | c |
| IFNG | 1.40 | 1.32 | c |
| IL12RB2 | 1.03 | 1.08 | c |
| IL13 | 1.50 | 1.61 | a |
| IL17F | 1.41 | 1.34 | a |
| IL17RB | 1.56 | 1.68 | a |
| IL18R1 | 0.86 | 0.86 | c |
| IL2RA | 1.38 | 1.30 | a |
| IL3 | 0.97 | 1.23 | c |
| IL5 | 2.20 | 2.32 | a |
| IL9 | 0.95 | 1.68 | c |
| ITGA2 | 1.05 | 0.50 | b |
| LIF | 1.65 | 1.56 | a |
| LTA | 1.20 | 1.25 | a |
| MS4A4A | -1.30 | -1.19 | c |
| OSM | 0.86 | 0.81 | c |
| SERPING1 | 0.63 | 1.04 | c |
| TNFSF4 | 1.04 | 0.79 | c |
| **Cell cycle/cell division** |  |  |  |
| ANLN | 1.17 | 0.89 | a |
| ASPM | 1.20 | 1.03 | a |
| AURKA | 0.83 | 0.74 | b |
| AURKB | 1.00 | 0.92 | c |
| BIRC5 | 1.02 | 0.78 | a |
| BUB1 | 0.90 | 0.76 | a |
| BUB1B | 0.91 | 0.86 | c |
| CASC5 | 1.02 | 1.13 | c |
| CCNA2 | 1.32 | 1.15 | a |
| CCNB1 | 1.05 | 0.93 | a |
| CCNB2 | 1.05 | 0.70 | a |
| CDC2 | 1.05 | 0.78 | b |
| CDC20 | 0.94 | 0.81 | a |
| CDC25A | 1.01 | 0.83 | a |
| CDC45L | 0.84 | 0.82 | b |
| CDCA2 | 1.02 | 0.86 | c |
| CENPE | 0.81 | 0.82 | a |
| CENPF | 0.83 | 0.77 | c |
| CEP55 | 1.14 | 0.85 | a |
| CHEK1 | 0.95 | 0.86 | a |
| CIT | 0.70 | 0.81 | c |
| CLSPN | 0.93 | 0.82 | c |
| DLG7 | 1.15 | 0.90 | a |
| DUB3 | 1.22 | 1.14 | a |
| ESCO2 | 1.01 | 0.84 | a |
| ESPL1 | 0.98 | 0.96 | a |
| GTSE1 | 0.95 | 0.99 | a |
| HJURP | 1.01 | 1.02 | a |
| KIF11 | 0.98 | 0.90 | a |
| MKI67 | 1.32 | 1.12 | a |
| NCAPG | 1.19 | 0.94 | a |
| NCAPH | 1.01 | 0.85 | a |
| NEK2 | 0.75 | 0.73 | b |
| NUF2 | 0.94 | 0.67 | b |
| NUSAP1 | 0.76 | 0.65 | b |
| PBK | 0.95 | 0.94 | c |
| PLK1 | 1.02 | 1.09 | a |
| RAD51 | 0.78 | 0.79 | c |
| SPBC25 | 0.91 | 1.05 | a |
| TPX2 | 1.37 | 1.13 | a |
| TRIP13 | 0.77 | 0.84 | c |
| TYMS | 1.21 | 1.05 | a |
| UBE2C | 1.04 | 0.58 | a |
| ANLN | 1.17 | 0.89 | a |
| ASPM | 1.20 | 1.03 | a |
| AURKA | 0.83 | 0.74 | b |
| AURKB | 1.00 | 0.92 | c |
| BIRC5 | 1.02 | 0.78 | a |
| BUB1 | 0.90 | 0.76 | a |
| BUB1B | 0.91 | 0.86 | c |
| CASC5 | 1.02 | 1.13 | c |
| CCNA2 | 1.32 | 1.15 | a |
| CCNB1 | 1.05 | 0.93 | a |
| CCNB2 | 1.05 | 0.70 | a |
| CDC2 | 1.05 | 0.78 | b |
| CDC20 | 0.94 | 0.81 | a |
| CDC25A | 1.01 | 0.83 | a |
| CDC45L | 0.84 | 0.82 | b |
| CDCA2 | 1.02 | 0.86 | c |
| CENPE | 0.81 | 0.82 | a |
| CENPF | 0.83 | 0.77 | c |
| CEP55 | 1.14 | 0.85 | a |
| CHEK1 | 0.95 | 0.86 | a |
| CIT | 0.70 | 0.81 | c |
| CLSPN | 0.93 | 0.82 | c |
| DLG7 | 1.15 | 0.90 | a |
| DUB3 | 1.22 | 1.14 | a |
| ESCO2 | 1.01 | 0.84 | a |
| ESPL1 | 0.98 | 0.96 | a |
| GTSE1 | 0.95 | 0.99 | a |
| HJURP | 1.01 | 1.02 | a |
| KIF11 | 0.98 | 0.90 | a |
| MKI67 | 1.32 | 1.12 | a |
| NCAPG | 1.19 | 0.94 | a |
| NCAPH | 1.01 | 0.85 | a |
| NEK2 | 0.75 | 0.73 | b |
| NUF2 | 0.94 | 0.67 | b |
| NUSAP1 | 0.76 | 0.65 | b |
| PBK | 0.95 | 0.94 | c |
| PLK1 | 1.02 | 1.09 | a |
| RAD51 | 0.78 | 0.79 | c |
| SPBC25 | 0.91 | 1.05 | a |
| TPX2 | 1.37 | 1.13 | a |
| TRIP13 | 0.77 | 0.84 | c |
| TYMS | 1.21 | 1.05 | a |
| UBE2C | 1.04 | 0.58 | a |
| **Cytoskeleton/microtubule** |  |  |  |
| CKAP2L | 1.04 | 0.91 | c |
| DIAPH3 | 0.82 | 0.75 | b |
| KIF14 | 0.98 | 0.86 | c |
| KIF15 | 1.08 | 0.69 | b |
| KIF18A | 0.77 | 0.78 | a |
| KIF23 | 1.11 | 0.80 | a |
| KIF2C | 1.04 | 0.89 | a |
| KIF4A | 0.89 | 0.89 | a |
| PRC1 | 1.02 | 0.91 | c |
| WIPF2 | 1.21 | 1.12 | a |
| **Chromosomal protein/DNA packaging** | | |  |
| CHAF1B | 0.74 | 0.72 | c |
| HIST1H2AB | 0.80 | 0.57 | c |
| HIST1H2AI | 0.74 | 0.68 | c |
| HIST1H2AJ | 1.01 | 0.92 | c |
| HIST1H2AL | 0.89 | 0.85 | c |
| HIST1H2BF | 0.76 | 0.78 | b |
| HIST1H2BJ | 0.76 | 0.67 | a |
| HIST1H2BL | 0.62 | 0.39 | b |
| HIST1H2BM | 1.39 | 1.17 | a |
| HIST1H2BN | 0.53 | 0.60 | a |
| HIST1H3B | 1.35 | 1.09 | a |
| HIST1H3F | 1.43 | 1.03 | c |
| HIST1H3G | 1.26 | 1.04 | b |
| HIST1H3H | 0.76 | 0.66 | a |
| HIST1H3J | 1.06 | 0.72 | c |
| HIST1H4F | 0.64 | 0.57 | a |
| HIST1H4I | 0.51 | 0.59 | a |
| HIST1H4L | 0.51 | 0.72 | a |
| HIST2H2AB | 0.85 | 0.64 | a |
| HIST2H3C | 1.19 | 0.88 | a |
| HISTH1A | 1.13 | 0.81 | b |
| HISTH1B | 1.21 | 0.97 | c |
| **DNA replication** |  |  |  |
| DTL | 1.24 | 1.13 | a |
| MCM10 | 1.14 | 0.89 | a |
| MCM4 | 0.77 | 0.71 | c |
| NEIL3 | 0.75 | 0.66 | c |
| ORC1L | 0.81 | 0.65 | a |
| RRM2 | 1.38 | 1.15 | a |
| TK1 | 1.04 | 0.65 | a |
| TOP2A | 1.16 | 0.98 | a |
| **Transcription regulation** |  |  |  |
| DUX2 | 1.18 | 1.23 | a |
| DUX4C | 1.18 | 1.23 | a |
| E2F7 | 0.88 | 0.81 | a |
| E2F8 | 0.88 | 0.59 | c |
| FOXM1 | 0.84 | 0.96 | c |
| PHF1 | 1.04 | 0.90 | a |
| SIX1 | 0.47 | 0.61 | c |
| SSX4 | 0.52 | 0.56 | c |
| TCEB3C | 0.69 | 0.83 | c |
| ZBTB32 | 0.57 | 0.66 | c |
| **Signal transduction** |  |  |  |
| ARHGAP11A | 0.81 | 0.77 | b |
| C9orf100 | 0.70 | 0.34 | b |
| DEPDC1B | 1.03 | 0.83 | a |
| DUSP4 | 1.03 | 0.72 | b |
| OR2T8 | 0.46 | 0.66 | c |
| OR6B3 | 0.91 | 0.58 | b |
| PCDHB2 | 0.74 | 0.76 | a |
| RAB19B | 0.57 | 0.72 | c |
| RP1L1 | 0.66 | 0.35 | b |
| SHC4 | 0.48 | 0.68 | c |
| **Other & non-classified** |  |  |  |
| APOL4 | 1.24 | 1.11 | c |
| AQP7 | 0.48 | 0.52 | c |
| C13orf3 | 0.88 | 0.84 | a |
| C15orf42 | 0.98 | 0.85 | c |
| C16orf59 | 0.75 | 0.72 | c |
| C18orf24 | 1.33 | 0.74 | a |
| ELOVL6 | 0.85 | 0.65 | c |
| FAM54A | 0.91 | 0.72 | b |
| FAM86A | 0.76 | 0.84 | c |
| FAM90A1 | 0.78 | 0.86 | c |
| FAM90A2P | 1.62 | 1.70 | a |
| HII-52 | 1.22 | 1.12 | a |
| IDI2 | 0.78 | 1.00 | c |
| KDELC1 | 1.09 | 0.51 | b |
| KIAA0101 | 1.09 | 0.81 | b |
| MELK | 1.15 | 0.94 | a |
| MERTK | -0.97 | -1.05 | c |
| MGC13005 | 0.66 | 0.84 | a |
| MLC1 | 0.61 | 0.71 | c |
| MMP12 | 1.46 | 1.61 | a |
| OR7E37P | 0.57 | 0.58 | b |
| PRAMEF8 | 0.89 | 0.73 | a |
| PRR11 | 0.93 | 0.81 | a |
| PSG5 | 0.78 | 0.56 | b |
| REXO1L1 | 1.26 | 1.08 | a |
| RP4-742C19.3 | 0.72 | 0.62 | b |
| SLC27A2 | 1.20 | 1.18 | a |
| ZBED2 | 0.66 | 0.67 | c |

Microarray gene expression profiles of CRM_197_-stimulated and unstimulated (control; CTLR) PBMC were compared for children in the neonatal (n = 25; pooled in groups of 5) and infant group (n = 25; pooled in groups of 5). Listed in functional clusters are the genes that were significantly differential expressed in response to CRM_197_ in one or both vaccination groups (SAM analysis, with false discovery rate (FDR) < 0.05 in groups (a), the neonatal group only (b), or the infant group only (c)). For each gene, the mean of the log_2_-fold differential expression is summarized for the neonatal and infant group.
