# Supplementary material for: Pneumococcal conjugate vaccination at birth in a high-risk setting: No evidence for neonatal T-cell tolerance
Source: Vaccine. 2011 Jul 26;29(33-19):5414–20. doi: 10.1016/j.vaccine.2011.05.065 (PMC3146700; doi:10.1016/j.vaccine.2011.05.065)
Supplement: Supplementary file 2 [file mmc2.ppt]

## Slide 1
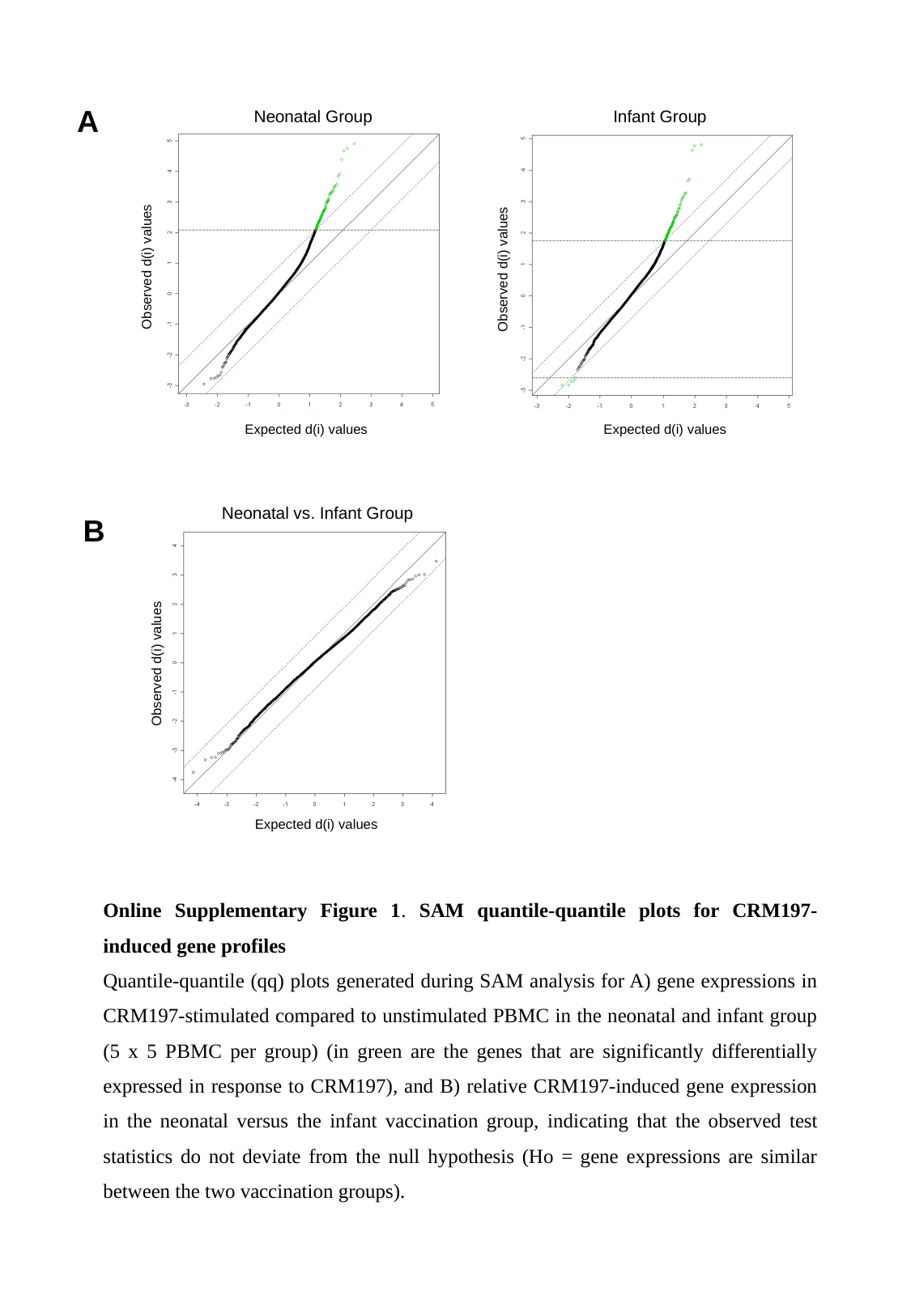

A
Neonatal Group
Infant Group
Observed d(i) values
Observed d(i) values
Expected d(i) values
Expected d(i) values
Neonatal vs. Infant Group
B
Observed d(i) values
Expected d(i) values
Online Supplementary Figure 1. SAM quantile-quantile plots for CRM197-induced gene profiles
Quantile-quantile (qq) plots generated during SAM analysis for A) gene expressions in CRM197-stimulated compared to unstimulated PBMC in the neonatal and infant group (5 x 5 PBMC per group) (in green are the genes that are significantly differentially expressed in response to CRM197), and B) relative CRM197-induced gene expression in the neonatal versus the infant vaccination group, indicating that the observed test statistics do not deviate from the null hypothesis (Ho = gene expressions are similar between the two vaccination groups).
